# Effects of Different Dietary Selenium Sources on the Meat Quality and Antioxidant Capacity of Yellow Catfish (*Pelteobagrus fulvidraco*)

**DOI:** 10.1155/2023/7981183

**Published:** 2023-07-29

**Authors:** Zheng Chen, Haokun Liu, Cui Liu, Shuzhan Fei, Xiaomin Hu, Dong Han, Junyan Jin, Yunxia Yang, Xiaoming Zhu, Shouqi Xie

**Affiliations:** ^1^State Key Laboratory of Freshwater Ecology and Biotechnology, Institute of Hydrobiology, Chinese Academy of Sciences, Wuhan 430072, China; ^2^University of Chinese Academy of Sciences, Beijing 100049, China; ^3^The Innovative Academy of Seed Design, Chinese Academy of Sciences, Beijing 100101, China

## Abstract

To assess the effect of dietary selenium (Se) sources on the meat quality and antioxidant capacity of yellow catfish (*Pelteobagrus fulvidraco*), sodium selenite (Na_2_SeO_3_), Se yeast, and selenium-enriched *Spirulina platensis* (Se–SP) were supplemented in the control diet at 0.30 mg Se/kg feed to formulate four diets. The experimental period lasted 50 days. The results showed that Se levels in the plasma, liver, muscle, and whole body were significantly increased by dietary Se yeast supplementation (*P* < 0.05) but showed no change in response to Na_2_SeO_3_ (*P* > 0.05). The three types of Se all increased the firmness and decreased the fracturability of the muscles (*P* < 0.05), but only Na_2_SeO_3_ resulted in higher springiness, flexibility, stringiness, and stickiness (*P* < 0.05). In addition, the muscle *n*−3 polyunsaturated fatty acid (PUFA) content was increased by Se yeast (*P* < 0.05). Regarding antioxidant capacity, dietary Se yeast and Se–SP supplementation improved hepatic glutathione peroxidase activity but decreased hepatic malondialdehyde content (*P* < 0.05). Given these results, Se yeast was found to be the optimal source of Se for yellow catfish for higher tissue retention, antioxidant capacity, and PUFA levels. Dietary Se is an effective way to regulate the meat quality and antioxidant capacity of yellow catfish.

## 1. Introduction

Se plays a key role in glutathione peroxidase (GPX), which can reduce oxidative damage and prevent lipid peroxidation in organisms, including humans and fish [1]. In addition to antioxidant properties, Se is also related to thyroid hormone synthesis, inflammation, and the immune response [2]. Insufficient or excessive Se in the diet may retard the growth and health of fish. Se requirements have been estimated for some freshwater fish and marine fish, including grouper (*Epinephelus malabaricus*) [3], channel catfish (*Ictalurus punctatus*) [4], rainbow trout (*Oncorhynchus mykiss*) [5–7], gibel carp (*Carassius auratus gibelio*) [8, 9], and Atlantic salmon (*Salmo salar*) [10].

The utilization of efficient and safe Se sources as aquatic feed additives to ensure optimal growth and health of farmed fish and to produce Se-enriched aquatic products is one of the themes of aquatic nutrition research. Various forms of Se, such as sodium selenite, selenate, Se yeast, selenomethionine, and nano-Se, are widely used as feed additives for aquaculture. There have been some comparative studies on bioavailability [11], acute toxicity [12], immunity [11], and other effects of different Se sources on fish. Organic Se accumulates more efficiently in tissues than inorganic Se in Atlantic salmon [13], grouper [3], and channel catfish [11].

Meat quality is an important factor of consumer choice. Dietary Se has been found to improve livestock and poultry meat quality [14–17]. However, little research has examined its effects on fish quality. Lin discovered that both selenomethionine and Na_2_SeO_3_ could enhance the meat quality of juvenile grouper [3]. In muscle foods, the meat quality attributes include characteristics such as texture, nutritional value, and appearance [18, 19]. Therefore, we determined the body color, body composition, fatty acid profile, and amino acid composition of muscle, measured tissue Se concentrations, and tested muscle texture characteristics. In addition to its indispensable role in the health of fish, Se is essential for human health. Se deficiency has been confirmed to be related to a series of chronic conditions, including Kashin–Beck disease, Keshan disease, and cardiovascular diseases such as atherosclerosis [20]. Supplementation of Se in the diet to maintain an optimal Se status may protect against cancer and improve general health in patients [21, 22]. Thus, Se-enriched fish meat can be a good option for humans to supplement Se.

Seventy-five percent of global edible aquaculture production comes from freshwater fish, and the production of freshwater fish has grown rapidly in recent decades [21]. Furthermore, there is robust aquaculture production and a good potential market for yellow catfish as a kind of high-quality freshwater aquatic food in China and East Asia. It is also one of the main farmed species for pond farming and cage aquaculture due to well-developed farming and breeding techniques and because of its desirable taste and tender meat without intermuscular bones [23]. However, little research has been done to compare the effect of different Se sources on the meat quality of yellow catfish. In this study, Na_2_SeO_3_, Se yeast, and Se–SP were supplemented into the yellow catfish diets and the growth performance, meat quality and antioxidant properties of the fish were determined. The present study may provide guidance for aquaculture to produce high-quality Se-rich fish meat.

## 2. Materials and Methods

### 2.1. Experimental Diets

Four isonitrogenous and isoenergetic diets (protein: 43% in dry matter) were formulated. No additional Se was supplemented in the control diet (Table 1), while three sources of Se, including sodium selenite (Na_2_SeO_3_, ≥97.0%, Sinopharm Chemical Reagent Co., Ltd., Shanghai, China), Se yeast (2338 mg Se/kg, Angel Yeast Co., Ltd., Yichang, China), and Se-enriched *Spirulina platensis* (Se–SP, 114 mg/kg), were accurately weighed, and then supplemented in the basic diet at 0.30 mg Se/kg feed. The three types of Se were added to the mineral premix first to ensure homogeneous distribution in each feed. Se levels were determined to be 0.79, 1.08, 1.14, and 1.12 mg/kg feed in the control diet, Na_2_SeO_3_ diet, Se yeast diet, and Se–SY diet, respectively. All ingredients were accurately weighed according to the dry weight, then were milled, sieved, added to water, stirred evenly, and then pelleted by a granulator. Until use, pellets were kept at 4°C after being dried to moisture less than 10%.

### 2.2. Feeding Trial

Yellow catfish were obtained from Dengjiazhou fish farm (Wuhan, China), then transported to Laohe fish farm (Jingzhou, China), and maintained in outdoor cages for 20 days to acclimate. Following 1 day of fasting for all fish, 360 healthy fish with a similar weight of 49.79 ± 0.65 g were selected and randomly assigned into 12 floating cages (1 × 1 × 1 m^3^). There were 30 fish in each cage, with three cages in each treatment. Fish were fed to apparent satiation twice daily at 7:30 and 17:30, and feed intake was recorded every day throughout the feeding trial, which lasted 50 days. Conditions were kept stable and suitable for yellow catfish during the whole feeding trial (water temperature 33.0 ± 4.0°C; dissolved oxygen content >6.5 mg/L; total ammonia <0.1 mg/L).

### 2.3. Sample Collection

Fish were fasted 24 hr before being sampled. After all, fish were weighed and counted, nine fish from each cage were selected randomly for sample collection and were euthanized by 60 mg/L MS-222 (Sigma, St Louis, MO, USA). From each group of nine, two fish were frozen at −20°C for whole-body proximate analysis and Se content determination, and two fish were tagged and brought back to the laboratory for skin color determination and muscle texture characteristics testing. Moreover, the body length and weight of three fish were measured to obtain the condition factor (CF), and then their viscera were dissected and weighed to obtain the viscerosomatic index (VSI). The remaining two fish were dissected after blood samples were collected by heparinized syringes. Then, muscle and liver tissues were quick-frozen in a −80°C refrigerator for enzyme activity and gene expression determination. Plasma samples were obtained by centrifugation of collected blood samples (3,500 rpm, 15 min, 4°C) and then stored at −80°C for analysis of enzyme activities, hormone levels, and biochemical indices.

### 2.4. Laboratory Analysis of Feed and Body Composition

The AOAC methods [24] were used to determine the proximate composition of two fish from each cage and four experimental feeds. These fish and feeds were dried in an electric thermostatic oven at 105°C to measure moisture levels. Then crude lipid and protein levels were determined using a Soxtec™ 2055 (Tecator™ Technology, Foss, Hilleroed, Denmark) and a KD-310-Autoanalyzer (Opsis, Furulund, Sweden), respectively. A muffle furnace (KS-12D-16A, Yingshan Jianli Electric Furnace Manufacturing Co., Ltd., Huangang, China) was used to measure the ash content (12 hr, 550°C) and a PARR1281 calorimeter (Parr Instrument Co., Moline, IL, USA) was used to measure the gross energy of the feed.

The Se levels of feeds, whole fish, vertebrae, liver, and muscle were measured by a hydride atomic absorption spectrophotometer (GB 5009.93-2017, PRC National Standard), while the plasma Se concentrations were measured by ICP–MS (NexlON300X, PekinElmer Co., Waltham, MA, USA). Plasma biochemical indices were collected by a BS-460 automatic biochemical analyzer (Shenzhen Mindray Bio-Medical Electronics Co., Ltd., Shenzhen, China), and Beijing North Institute of Biological Technology's (Beijing, China) assay kits were used to measure plasma hormone levels. An A300 amino acid analyzer (membraPure, Hennigsdorf, Germany) was used to examine the levels of amino acids in the muscle after being freeze-dried and then hydrolyzed for 24 hr at 110°C according to the method of Fei et al. [25]. Fatty acid analysis was conducted on the muscle tissue using GC–MS (7890A, Agilent Technologies, Santa Clara, CA, USA) according to the methods of AOAC [26].

### 2.5. Determination of Fish Skin Color and Muscle Texture Characteristics

The skin color indices of the dorsal and abdomen were collected by a photoelectric tristimulus colorimeter (CR-400, Minolta, Osaka, Japan) and represented as *L*, *a* ^*∗*^ and *b* ^*∗*^ values as recommended by the International Commission on Illumination [27]. Afterward, the fish muscle tissue samples were dissected to measure the muscle texture characteristics using a texture analyzer (TA. XT plus, Stable Micro Systems Ltd., Godalming, UK). The TPA described by Bourne MC [28] is often used for texture analyses, but the size of the sliced fish fillets affects the results for the texture characteristics. Therefore, we made some modifications to the experimental method, and the fish were cut along the spine from the middle to obtain the fillets. Then, a P/2 N −2 mm diameter stainless steel needle probe was chosen to test skin strength and adhesiveness according to the method adapted from the ASTM Standard Method of Test D1321-95. For firmness and springiness, F2 (the force after 60 s) and F1 (the maximum force) were determined by a P/20 −20 mm diameter cylinder probe. Firmness can be measured by applying pressure to a preset distance on a fillet. To obtain the value for springiness, F2 is divided by F1 and then multiplied by 100%. We also used a P/20 −20 mm diameter cylinder probe and the method adapted from PEAR1/P2 to obtain the flexibility, fracturability, stringiness, and stickiness of the fish fillets. The measurement described here provides an alternative method of measuring texture characteristics using the TA. XT plus.

### 2.6. Determination of Malondialdehyde (MDA) and Antioxidant Enzyme Activity

The activities of superoxide dismutase (SOD) and GPX, the levels of MDA in the liver and plasma, and the activities of glutathione reductase (GR) and catalase (CAT) in the liver were determined by assay kits (Nanjing Jiancheng Bioengineering Institute, Nanjing, China).

### 2.7. Total RNA Extraction, cDNA Synthesis, and Quantitative RT-PCR

TRIzol reagent (Invitrogen, Carlsbad, CA, USA) was used to extract total RNA from the liver samples. After ensuring purity and integrity, the concentration was measured, and 1 *μ*g of extracted RNA was added to the reaction system for reverse transcription using a synthesis kit (M-MLV FirstStrand, Invitrogen). Real-time quantitative polymerase chain reaction was performed on a LightCycle 480 II system (Roche, Basel, Switzerland), and Table 2 lists all primers used in the real-time PCR system for which *β*-actin was selected as a housekeeping gene. The PCR program was set as follows: 5 min preincubation at 95°C, 40 cycles with 10 s at 95°C, 20 s at melting temperature, and 10 s at 75°C. Based on the methods of Vandesompele et al. [29], relative expression levels were calculated.

### 2.8. Statistical Analysis

One-way analysis of variance was carried out after all the data had been subjected to tests for normality and homogeneity of variance, which were performed on SPSS 25.0 (IBM, Chicago, IL, USA). Duncan's multiple range test was used to detect significant differences between groups, and the significance level was set at *P* < 0.05. The correlation analysis followed the methods described by Fei et al. [30].

## 3. Results

### 3.1. Growth Performance and Morphological Parameters

As shown in Table 3, no change in growth and feed conversion indices, including final body weight (FBW), specific growth rate (SGR), and feed efficiency (FE), was observed (*P* > 0.05). The Na_2_SeO_3_ and Se yeast groups showed significantly higher feeding rates (FRs), and dietary Se yeast and Se–SP supplementation resulted in higher CFs (*P* < 0.05). However, no difference in the VSI was observed (*P* > 0.05).

### 3.2. Body Composition and Se Accumulation

**Figure 1:**
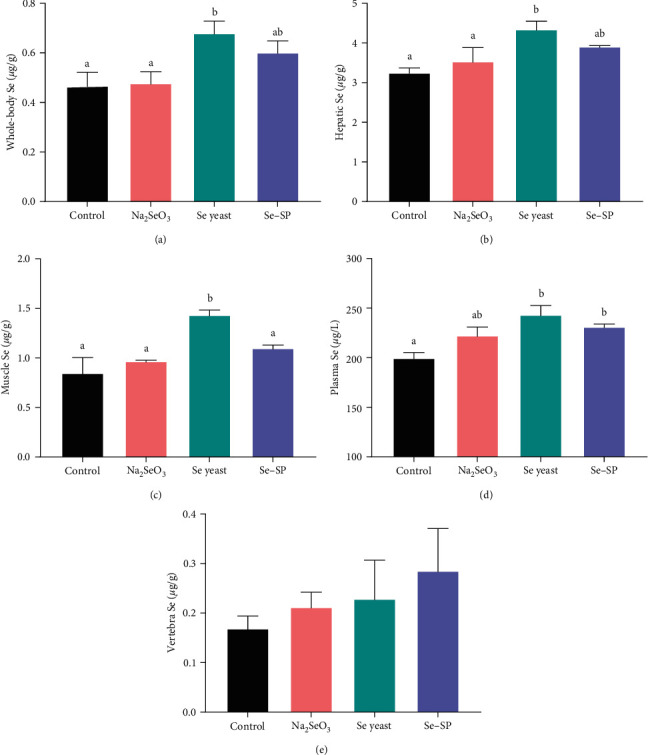
Total selenium concentrations: (a) whole body, (b) liver, (c) muscle, (d) plasma, and (e) vetabra of yellow catfish fed different experimental diets. Values are expressed as the means ± SEs (*n* = 3); different letters represent statistically significant differences (*P* < 0.05).

For whole-body composition, only the crude lipid content of fish from the Na_2_SeO_3_ group was significantly higher than that of fish from the Se yeast group (*P* < 0.05) (Table 4). There were significant increases in the whole-body, hepatic, and muscle Se levels of fish in the Se yeast group compared with both the control group and Na_2_SeO_3_ group (*P* < 0.05) (Figure 1). Fish in the Se yeast and Se–SP groups showed significantly higher plasma Se levels than fish in the control group, but only Se yeast increased muscle Se levels significantly (*P* < 0.05). There was no significant difference in the vertebra Se content among all treatments (*P* > 0.05).

### 3.3. Plasma Biochemistry and Hormone Levels

Plasma biochemical indices and hormone levels are shown in Table 5. TP, TC, LDL-C, GLU, INS, COR, TSH, and FT4 levels showed no significant difference among all groups (*P* > 0.05). Fish in the Se–SP group had lower plasma TG content than those in the Na_2_SeO_3_ group and lower plasma FT3 content than those in the Se yeast group (*P* < 0.05). In addition, a significant increase in plasma HDL-C levels was observed in the Se yeast and Se–SP groups (*P* < 0.05).

### 3.4. Body Color

The *b* ^*∗*^ values of the dorsal skin were significantly reduced by Na_2_SeO_3_ supplementation, while the *a* ^*∗*^ values of the dorsal skin were significantly reduced by yeast selenium supplementation (*P* < 0.05) (Table 6). Other values showed no significant difference (*P* > 0.05).

### 3.5. Main Amino Acid Analysis and Fatty Acid Profiles of Muscle

The muscle amino acid analysis and fatty acid composition of all treatments are presented in Tables 7 and 8. The Na_2_SeO_3_ diet significantly reduced the levels of arginine, valine, and aspartic acid in the fish muscles, while the Se yeast diet increased muscle proline levels (*P* < 0.05). The Se yeast group showed significantly lower muscle SFA levels due to lower C18 : 0 contents (*P* < 0.05). Similarly, the Se yeast group exhibited lower MUFA levels than the Na_2_SeO_3_ group due to lower C18 : 1 levels (*P* < 0.05). In contrast, EPA levels were found to be significantly higher in the Se yeast group than in the Na_2_SeO_3_ and Se–SP groups (*P* < 0.05). In addition, the Se yeast group exhibited the highest levels of DHA and *n*−3 polyunsaturated fatty acids (PUFAs), as well as *n*−3/*n*−6 ratios (*P* < 0.05).

### 3.6. Muscle Texture Characteristics

Table 9 illustrates the effects of different Se sources on the muscle texture characteristics of yellow catfish. The three types of dietary Se increased the firmness but decreased the fracturability significantly (*P* < 0.05). The skin strength and adhesiveness showed no differences (*P* > 0.05). However, the Na_2_SeO_3_ group showed significantly higher springiness, flexibility, stringiness, and stickiness than the control group (*P* < 0.05).

### 3.7. Antioxidant Enzymes and MDA

As shown in Table 10, dietary Se yeast and Se–SP significantly improved liver GPX activity, but only Se yeast significantly improved plasma GPX activity (*P* < 0.05). Plasma MDA levels decreased significantly with dietary Se supplementation (*P* < 0.05). No changes in hepatic GR, hepatic SOD, plasma SOD, and plasma MDA were found (*P* > 0.05).

### 3.8. Gene Expression

**Figure 2:**
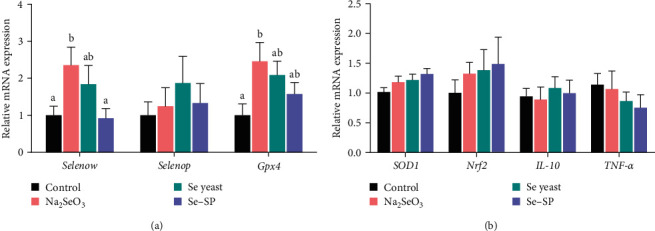
Gene expression of *selenow*, *selenop*, and *Gpx4* (a) and *SOD-1*, *Nrf2*, *IL-10*, and *TNF-α* (b) in the livers of yellow catfish fed different diets. *Selenow*, selenoprotein W; *selenop*, selenoprotein P; *Gpx4*, glutathione peroxidase 4; *SOD1*, superoxide dismutase; *Nrf2*, nuclear factor erythroid-2-related factor 2; *IL-10*, interleukin 10; *TNF-α*, tumor necrosis factor alpha. Values are expressed as the means ± SEs (*n* = 6) and expressed as the ratio of each gene relative to the expression of the housekeeping gene *β*-actin; different letters represent statistically significant differences (*P* < 0.05).

As shown in Figure 2(a), the hepatic expression of *selenow* and *gpx4* was upregulated in the Na_2_SeO_3_ group compared with the control group (*P* < 0.05). Genes expression levels involved in immune and antioxidant defense in the liver are shown in Figure 2(b). The expression of *sod-1*, *Nrf2*, *il-10*, and *tnf-α* was not affected by different Se sources (*P* > 0.05).

### 3.9. Correlation Analysis

**Figure 3:**
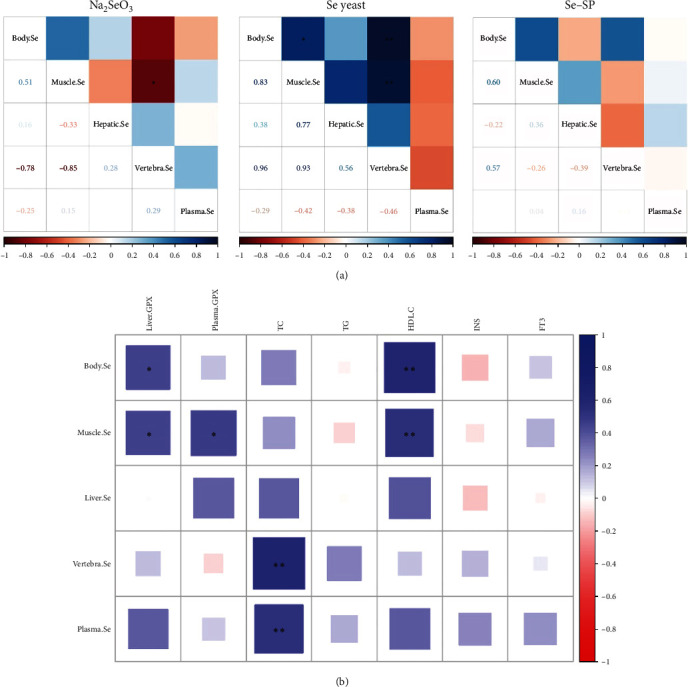
(a) Correlation plot of Se concentrations in different tissues of yellow catfish; (b) correlation plot between antioxidant capacity and plasma biochemical parameters and tissue Se concentration of yellow catfish. The color of the boxes demonstrates the level of the correlation index. Each significant parameter is marked with  ^*∗∗*^(*P* < 0.01) and  ^*∗*^(*P* < 0.05).

A correlation plot of Se concentrations in different tissues is presented in Figure 3(a). The vertebra Se concentration was significantly negatively correlated with that in muscle in the Na_2_SeO_3_ group (*P* < 0.05). However, the vertebra Se concentration was significantly positively correlated with that in muscle and whole body in the Se–SP group (*P* < 0.01). The muscle Se concentration was significantly positively correlated with that in the whole body in the Se–SP group (*P* < 0.05). As shown in Figure 3(b), liver GPX activity was significantly positively correlated with whole body and muscle Se concentrations, and plasma GPX activity was significantly positively correlated with muscle Se concentrations (*P* < 0.05). In addition, the plasma TC content was significantly positively correlated with the vertebra and plasma Se concentrations (*P* < 0.01), and the plasma HDL-C content was significantly positively correlated with the whole body vertebra and muscle Se concentrations (*P* < 0.01).

## 4. Discussion

Both inorganic (Na_2_SeO_3_) and organic (Se yeast and Se–SP) Se sources were supplemented into yellow catfish diets to assess their effects on fish growth performance, but neither positive nor negative effects were found. Deficient or excess dietary Se may retard the growth of fish even though deficiencies are unlikely to occur [10, 31]. For fingerling channel catfish, the dietary Se requirement should be no less than 0.25 mg/kg, and excess dietary Se at 15 mg/kg was shown to cause toxicity for growth [32]. In addition, when rainbow trout consume 1.25 mg/kg Se, the fish can still maintain homeostasis, but when the fish's long-term intake of Se exceeds 3 mg/kg, chronic dietary Se toxicity may occur [6]. It can be inferred that 0.79 mg/kg Se in the basal diet already meets the requirement of yellow catfish for Se, and supplementation with 0.30 mg/kg Se from dietary Na_2_SeO_3_, Se yeast, or Se–SP for 50 days did not cause toxicity because no depressed growth performance was observed in this study.

Metabolic pathways of different sources of Se differ, as do their bioavailabilities [33]. Studies on yellowtail kingfish (*Seriola lalandi*) [34] and channel catfish [11] showed that organic Se was more available for growth than inorganic Se was, and Se yeast promoted growth more effectively than Na_2_SeO_3_. This finding revealed that organic Se and inorganic Se had similar effects on the growth and feed utilization of yellow catfish, which was consistent with those of crucian carp [8], rainbow trout fry [5], and grouper [3]. The cause of the difference may be species or body size variation. In addition, dietary organic Se supplementation may be an efficient way to improve CFs. Studies on crucian carp [8], gibel carp [9], and rainbow trout [5] showed that dietary Se had no effect on body composition. However, the hepatic metabolism of largemouth bass (*Micropterus salmoide*) was affected by high dietary Se concentrations [35]. In our study, the whole-body lipid levels were regulated by different Se sources, and further research is needed to investigate this issue.

Organic Se had higher bioavailability than inorganic forms and showed higher rates of accumulation in muscle in grouper [3] and channel catfish [4]. Our results are consistent with findings for Atlantic salmon [13], in which the Se yeast group had higher muscle Se concentrations than the Na_2_SeO_3_ group, and no increase in muscle Se concentration was observed by inorganic Se supplementation. Interestingly, this study found that the whole body Se, hepatic Se, muscle Se, and plasma Se levels were observably increased with the supplementation of dietary Se yeast but showed no change in response to dietary Na_2_SeO_3_ supplementation. A positive correlation of tissue Se concentrations was observed only in the Se yeast group. Notably, Se–SP can only be deposited in plasma. Due to the differences in metabolic pathways, organic Se in the forms of selenomethionine (Se–Met) and selenocysteine (Se–Cys) can be stored in the muscle, while inorganic Se cannot. In the present study, Se yeast accumulated effectively in the muscle tissue of yellow catfish, while inorganic Se (Na_2_SeO_3_) and Se–SP did not. Yellow catfish may be unable to convert inorganic Se into Se–Met or Se–Cys directly, and it is difficult for it to incorporate the Se of Se–SP into its protein. Se yeast may be rich in Se–Met, which has a long metabolic cycle and high bioavailability in vivo [36]. As a target organ of Se metabolism, the liver had high Se levels that showed the same pattern as the Se levels of the whole body. Tapiero et al. [20] found that chronic feeding of inorganic Se compounds can be hepatotoxic in animals. In our study, yellow catfish may have removed inorganic Se from the body through the detoxification function of the liver, such that there was hardly any conversion of absorbed inorganic Se into a biologically active form.

Plasma glucose, total protein, and total cholesterol did not change in response to dietary Se addition. These results are consistent with the findings for rainbow trout [37] but differ from those for *Pangasius hypophthalmus*, in which glucose and cortisol levels were elevated under stress conditions [12]. Se is also associated with the lipid metabolism of fish. For dietary Se concentrations, marginal or excess Se may increase TG concentrations in the intestine and increase lipid deposition in yellow catfish [38]. In the present study, plasma TC and HDL-C levels were positively correlated with tissue Se concentrations, which also indicated the ability of Se to regulate lipid metabolism. For different dietary Se sources, this finding suggests that Na_2_SeO_3_ may cause higher plasma TG levels and higher whole-body lipid levels. FT3 is the inactive metabolite T3 (triiodothyronine), and T3 is the biologically active form of thyroid hormone [39]. Se yeast resulted in higher FT3 in plasma, which means a higher metabolic rate. This may be the reason that Se yeast resulted in higher FRs. Additionally, lipoproteins are crucial for the transportation of THs [40]. Meanwhile, the plasma HDL-C showed the same pattern as the plasma Se concentrations, which was also consistent with the CF for yellow catfish.

With the development of the market for aquatic foods, consumers are paying increasing attention to the quality of aquatic foods. Fish quality includes safety, appearance, nutritional quality, texture, and other parameters [19]. Lin [3] found that neither organic nor inorganic Se has any effect on the color of grouper muscle. Compared with muscle color, skin color may be more susceptible to the influence of minor elements in feed additives. Some feed additives, such as *S. platensis*, have been reported to improve the skin color of yellow catfish [24]. Therefore, we evaluated the effect of Se on the skin color and found that supplementation with Na_2_SeO_3_ and Se yeast altered the skin color of yellow catfish but Se–SP did not. However, to verify this effect, further research is needed.

Several kinds of minor elements, including Se, as feed additives, could improve meat quality [3, 12, 41]. Our study demonstrated the negative effects of Na_2_SeO_3_ on the amino acid profiles and the increase in *n*−3 PUFA levels in yellow catfish muscle by dietary Se yeast supplementation. For juvenile grouper, both dietary organic and inorganic Se supplementation enhanced meat quality [3]. Some studies have suggested that Se supplementation could improve meat quality due to its ability to slow the oxidation of meat. We found that the firmness of fish could be effectively improved by dietary Se supplementation. In addition, the enhancement of springiness, flexibility, stringiness, and stickiness by dietary Na_2_SeO_3_ supplementation may be related to higher whole-body lipid levels.

GPX activity is a useful index for assessing the bioavailability of Se [42]. Our results were in line with studies on largemouth bass [35], juvenile grouper [42], and crucian carp [8], which showed higher hepatic GPX activity than the control. This result was different from the results found in channel catfish [4] and Atlantic salmon [13], which exhibited no change in hepatic GPX activity. As a kind of Se-containing enzyme, GPX is important in forming the antioxidant defense system against endogenous reactive oxygen species. We found that hepatic and plasma GPX activity had a positive correlation with muscle Se concentration and that dietary Se yeast supplementation resulted in higher muscle Se levels. This study also suggests that dietary Se supplementation, especially organic Se, could effectively improve hepatic GPX activity. As a marker of oxidative stress, hepatic MDA content had a negative correlation with hepatic GPX activities, and it decreased with three sources of Se supplementation. Only Se–SP could improve hepatic CAT activity, which may be due to the antioxidant capacity of *S. platensis* [24].

Different selenoproteins have different functions and thus show different transcriptional responses, and selenoproteins exhibit different patterns in different tissues [38, 39]. In yellow catfish, only seven selenoprotein genes have been characterized [43]. Studies on the hepatic expression of selenoproteins by dietary Se sources have yet to be published. Dietary Na_2_SeO_3_ supplementation was found to upregulate the expression of *gpx4* and *selenow*, which was inconsistent with the total Se accumulation and GPX activity in the liver. We only determined the hepatic expression of three selenoprotein genes. In addition, the results were in agreement with the findings of other studies indicating that changes in protein expression did not always correspond to changes in mRNA [44].

## 5. Conclusion

In conclusion, Se yeast seems to be the optimal Se source for yellow catfish for higher tissue retention, antioxidant capacity, and *n*−3 PUFA levels. Dietary supplementation with Se yeast is an efficient way to produce Se-enriched fish meat, which is a good source of Se for human health. Dietary Se is an effective way to regulate the meat quality and antioxidant capacity of yellow catfish.

## Figures and Tables

**Table 1:** Formulation and proximate composition of the basal diet (% in dry matter).

Ingredients	%
White fishmeal^1^	32.00
Soybean meal^2^	17.00
Rapeseed meal	17.55
Corn starch	9.73
Wheat flour	10.00
Fish oil	2.53
Soybean oil	2.53
Vitamin premix^3^	0.39
Mineral premix^4^	5.00
Choline chloride	0.11
Cellulose	0.16
Carboxymethyl cellulose	3.00
Proximate composition	
Crude protein	42.96
Crude fat	6.32
Ash	13.21
Moisture	7.99
Gross energy (kJ/g)	17.35

^1^White fish meal: purchased from American Seafood Company, Seattle, Washington, USA. ^2^Soybean meal: purchased from Wuhan Coland Feed Co. Ltd, Wuhan, Hubei, China. ^3^Vitamin premix (mg/kg diet): vitamin B_1_, 20; vitamin B_2_, 20; vitamin B_6_, 20; vitamin B_12_, 0.02; folic acid, 5; calcium patothenate, 50; inositol, 100; niacin, 100; biotin, 0.1; cellulose, 3412; ascorbic acid, 100; vitamin A, 11; vitamin D, 2; vitamin E, 50; vitamin K, 10. ^4^Mineral premix (mg/kg diet): NaCl, 500.0; MgSO_4_·7H_2_O, 8155.6; NaH_2_PO_4_·2H_2_O, 12,500.0; KH_2_PO_4_, 16,000.0; Ca(H_2_PO_4_) ·2H_2_O, 7,650.6; FeSO_4_·7H_2_O, 2,286.2; C_6_H_10_CaO_6_·5H_2_O, 1,750.0; ZnSO_4_·7H_2_O, 178.0; MnSO_4_·H_2_O, 61.4; CuSO_4_·5H_2_O, 15.5; CoSO_4_·7H_2_O, 0.91; KI, 1.5; corn starch, 900.3.

**Table 2:** Primers used for real-time PCR analysis.

Gene	Forwards primer (5′-3′)	Reverse primer (5′-3′)	Size (bp)	Accession no.
*selenow*	CGGTCGCTCGTGATGTGAT	AGCTCGGTTTAAAGGCAGGA	100	XM027178802.1
*selenop*	AGGCATGTAACAGGACCACG	GACTATGCCTCGCTCCACTC	156	XM027171023.1
*gpx4*	TGGTGTGCAAAACCTACCCC	TTAGTCTTCATCGCTACACGGT	122	XM027150431.1
*sod1*	TGCAGGACCTCACTTCAACC	TTCCATCGGAATCGGCAGTC	104	XM027171881.1
*il-10*	CTCCTCCCCCTGAGGATTCA	CGGATCACGGCGTATGAAGA	227	XM027144360.1
*tnf-α*	ATCAGGTGAACGCTGATGCT	GTGTTGAGGGAAGGGGTCTG	98	XM027142122.1
*Nrf2*	TCTCGCCCAGTTACAGCTTG	GTTCCGTGAACGCCACATTC	128	XM027164284.1
*β-actin*	TTCGCTGGAGATGATGCT	CGTGCTCAATGGGGTACT	160	XM027148463.1

Note. *selenow*, selenoprotein W; *selenop*, selenoprotein P; *gpx4*, glutathione peroxidase 4; *sod1*, superoxide dismutase; *il-10*, interleukin 10; *tnf-α*, tumor necrosis factor a; *Nrf2*, nuclear factor erythroid-2-related Factor 2.

**Table 3:** Effects of different dietary selenium sources on growth performance and morphological parameters of yellow catfish.

	Control	Na_2_SeO_3_	Se yeast	Se–SP
IBW (g)	49.89 ± 0.59	50.06 ± 0.47	49.67 ± 0.19	49.56 ± 0.29
FBW (g)	77.79 ± 2.63	85.29 ± 3.83	82.71 ± 3.63	80.23 ± 0.99
FR (%BW/d)	2.23 ± 0.02^a^	2.45 ± 0.07^bc^	2.60 ± 0.01^c^	2.36 ± 0.07^ab^
SGR (%/d)	0.92 ± 0.07	1.10 ± 0.10	1.04 ± 0.08	0.98 ± 0.02
FE (%)	39.94 ± 3.06	42.92 ± 3.05	37.76 ± 2.41	40.60 ± 1.81
CF (g/cm^3^)	1.53 ± 0.05^a^	1.73 ± 0.04^ab^	1.81 ± 0.06^b^	1.81 ± 0.11^b^
VSI (%)	9.80 ± 0.54	10.93 ± 0.53	10.56 ± 0.52	10.4 ± 0.63

Values are expressed as the means ± SEs; different letters represent statistically significant differences (*P* < 0.05). IBW, initial body weight; FBW, final body weight; FR, feeding rate (%BW/d) = 100 × dry feed intake/[days × (IBW + FBW)/2]; SGR, specific growth rate (%/d) = 100 × [ln (final body weight)−ln (initial body weight)]/days; FE, feed efficiency (%) = (final body weight-initial body weight)/feed intake in dry matter; CF, condition factor (g/cm^3^) = 100 × (body weight)/(body length)^3^; VSI, viscerosomatic index (%) = 100 × (viscera weight)/(whole body weight).

**Table 4:** Whole-body composition of yellow catfish fed different experimental diets (% dry matter).

	Control	Na_2_SeO_3_	Se yeast	Se–SP
Crude protein	14.95 ± 0.17	14.47 ± 0.37	15.12 ± 0.24	15.06 ± 0.26
Crude lipid	10.22 ± 0.43^ab^	10.87 ± 0.82^b^	9.07 ± 0.23^a^	9.40 ± 0.20^ab^
Ash	4.17 ± 0.05	4.09 ± 0.06	4.12 ± 0.10	4.36 ± 0.19
Moisture	70.26 ± 0.61	69.63 ± 1.5	71.06 ± 0.14	70.42 ± 0.63

Values are expressed as the means ± SEs; different letters represent statistically significant differences (*P* < 0.05).

**Table 5:** Effects of dietary supplementation of different selenium sources on plasma biochemical indices and plasma hormone levels in yellow catfish.

	Control	Na_2_SeO_3_	Se yeast	Se–SP
TP (g/L)	27.07 ± 1.48	28.75 ± 1.68	29.62 ± 0.82	29.20 ± 0.60
TG (mmol/L)	4.23 ± 0.41^ab^	6.38 ± 1.32^b^	4.22 ± 0.85^ab^	3.80 ± 0.68^a^
TC (mmol/L)	4.37 ± 0.21	4.99 ± 0.47	5.23 ± 0.31	5.14 ± 0.32
LDL-C (mmol/L)	1.46 ± 0.37	1.41 ± 0.19	1.41 ± 0.15	1.41 ± 0.14
HDL-C (mmol/L)	1.91 ± 0.11^a^	2.13 ± 0.11^ab^	2.37 ± 0.08^b^	2.32 ± 0.08^b^
GLU (mmol/L)	2.70 ± 0.58	2.63 ± 0.34	2.45 ± 0.38	2.71 ± 0.31
INS (*μ*IU/mL)	9.83 ± 1.68	9.82 ± 1.45	10.67 ± 1.39	11.29 ± 2.03
COR (*μ*g/L)	192.3 ± 19.86	218.05 ± 10.80	195.02 ± 17.43	192.24 ± 7.76
TSH (*μ*IU/mL)	0.27 ± 0.06	0.30 ± 0.11	0.24 ± 0.07	0.26 ± 0.06
FT3 (pmol/L)	5.86 ± 0.89^ab^	7.79 ± 0.84^ab^	9.11 ± 1.21^b^	4.67 ± 1.05^a^
FT4 (pmol/L)	0.49 ± 0.15	0.51 ± 0.18	0.55 ± 0.14	0.67 ± 0.15

TP, total protein; TG, triglyceride; TC, total cholesterol; LDL-C, low-density lipoprotein cholesterol; HDL-C, high-density lipoprotein cholesterol; GLU, glucose; INS, insulin; COR, cortisol; TSH, thyroid-stimulating hormone; FT3, free triiodothyronine; FT4, free thyroxine. Values are expressed as the means ± SEs (*n* = 6); different letters represent statistically significant differences (*P* < 0.05).

**Table 6:** Body color of yellow catfish fed different experimental diets.

	Control	Na_2_SeO_3_	Se yeast	Se–SP
Abdominal skin				
Lightness (*L*)	83.10 ± 1.21	81.82 ± 0.74	83.05 ± 0.55	81.92 ± 0.79
Redness (*a* ^*∗*^)	−11.25 ± 0.68	−10.71 ± 0.53	−12.39 ± 0.43	−10.36 ± 0.58
Yellowness (*b* ^*∗*^)	42.80 ± 3.79	48.67 ± 1.71	49.82 ± 3.57	41.05 ± 4.57
Dorsal skin				
Lightness (*L*)	35.02 ± 0.83	33.3 ± 0.98	32.93 ± 1.52	32.53 ± 1.65
Redness (*a* ^*∗*^)	−5.17 ± 0.14^b^	−4.39 ± 0.30^ab^	−4.08 ± 0.36^a^	−4.78 ± 0.25^ab^
Yellowness (*b* ^*∗*^)	13.71 ± 0.79^b^	11.35 ± 0.27^a^	13.19 ± 0.46^b^	13.72 ± 0.47^b^

Values are expressed as the means ± SEs (*n* = 6); different letters represent statistically significant differences (*P* < 0.05).

**Table 7:** Effects of different dietary selenium sources on the amino acid composition (% dry matter) of yellow catfish muscles.

	Control	Na_2_SeO_3_	Se yeast	Se–SP
EAA^1^				
Arg	4.11 ± 0.19^b^	3.49 ± 0.19^a^	4.25 ± 0.11^b^	4.07 ± 0.13^b^
His	1.63 ± 0.07^ab^	1.44 ± 0.11^a^	1.77 ± 0.05^b^	1.65 ± 0.05^ab^
Ile	2.88 ± 0.15	2.51 ± 0.21	2.95 ± 0.06	2.79 ± 0.11
Leu	5.37 ± 0.27	4.68 ± 0.35	5.57 ± 0.12	5.31 ± 0.21
Lys	6.36 ± 0.28	5.60 ± 0.37	6.49 ± 0.18	6.14 ± 0.18
Met	1.64 ± 0.12	1.56 ± 0.11	1.78 ± 0.11	1.64 ± 0.06
Phe	2.90 ± 0.14	2.42 ± 0.15	2.79 ± 0.09	2.74 ± 0.07
Thr	3.29 ± 0.15	2.89 ± 0.14	3.29 ± 0.08	2.85 ± 0.23
Val	3.17 ± 0.16^b^	2.72 ± 0.17^a^	3.31 ± 0.07^b^	3.10 ± 0.09^ab^
NEAA^2^				
Asp	7.08 ± 0.30^b^	5.99 ± 0.36^a^	6.84 ± 0.17^ab^	6.36 ± 0.22^ab^
Ser	3.20 ± 0.13	2.75 ± 0.18	3.13 ± 0.08	3.10 ± 0.10
Glu	10.43 ± 0.53	9.35 ± 0.6	11.33 ± 0.3	10.5 ± 0.51
Gly	3.44 ± 0.24	3.22 ± 0.18	3.88 ± 0.11	3.47 ± 0.09
Ala	4.24 ± 0.19	3.72 ± 0.2	4.2 ± 0.09	4.04 ± 0.11
(Cys)_2_	0.49 ± 0.04	0.48 ± 0.05	0.44 ± 0.04	0.46 ± 0.02
Tyr	2.26 ± 0.11	2.00 ± 0.19	2.43 ± 0.05	2.15 ± 0.11
Pro	1.69 ± 0.09^a^	1.69 ± 0.14^a^	2.11 ± 0.03^b^	1.79 ± 0.13^ab^
TAA^3^	64.17 ± 2.98	56.51 ± 3.52	66.55 ± 1.46	62.16 ± 2.00

Values are expressed as the means ± SEs (*n* = 6); different letters represent statistically significant differences (*P* < 0.05). ^1^EAA, essential amino acids. ^2^NEAA, nonessential amino acids. ^3^TAA, total amino acids.

**Table 8:** Effects of different dietary selenium sources on muscle main fatty acid profiles (% of total fatty acids) in yellow catfish.

	Control	Na_2_SeO_3_	Se yeast	Se–SP
C14 : 0	2.34 ± 0.09	2.11 ± 0.23	2.05 ± 0.13	2.38 ± 0.11
C16 : 0	11.42 ± 0.12	9.80 ± 1.29	9.81 ± 0.54	10.54 ± 0.79
C18 : 0	12.02 ± 0.54^b^	10.76 ± 0.8^ab^	9.22 ± 0.58^a^	11.21 ± 0.84^ab^
*Σ*SFA^1^	25.78 ± 0.45^c^	22.67 ± 0.87^ab^	21.07 ± 1.18^a^	24.13 ± 0.3^bc^
C16 : 1	3.09 ± 0.13	3.59 ± 0.38	3.50 ± 0.52	4.05 ± 0.94
C18 : 1	25.57 ± 0.70^ab^	32.03 ± 3.22^b^	21.06 ± 2.43^a^	26.89 ± 1.05^ab^
C20 : 1	4.00 ± 0.36	4.25 ± 0.59	5.66 ± 1.10	4.13 ± 0.20
C22 : 1	4.36 ± 0.22	3.43 ± 0.54	3.43 ± 0.14	3.58 ± 0.30
*Σ*MUFA^2^	37.02 ± 0.04^ab^	43.31 ± 2.96^b^	33.65 ± 2.35^a^	38.65 ± 0.9^ab^
C18 : 2*n*−6 (LA)	8.97 ± 0.20	11.51 ± 1.37	8.22 ± 0.70	9.14 ± 0.77
C20 : 4*n*−6 (ARA)	6.25 ± 0.14	5.31 ± 0.61	5.59 ± 0.19	5.45 ± 0.26
*Σn*−6 PUFA^3^	15.22 ± 0.12	16.82 ± 0.76	13.81 ± 0.58	14.59 ± 1.03
C18 : 3*n*−3 (LNA)	3.37 ± 0.19	3.16 ± 0.28	3.81 ± 0.24	3.39 ± 0.27
C20 : 5*n*−3 (EPA)	4.65 ± 0.32^ab^	4.04 ± 0.39^a^	5.71 ± 0.43^b^	4.36 ± 0.07^a^
C22 : 6*n*−3 (DHA)	13.96 ± 0.50^a^	10.01 ± 2.45^a^	21.96 ± 2.17^b^	14.89 ± 1.11^a^
*Σn*−3 PUFA^4^	21.98 ± 0.46^a^	17.21 ± 2.46^a^	31.48 ± 2.72^b^	22.64 ± 1.22^a^
*n*−3/*n*−6	1.44 ± 0.03^a^	1.03 ± 0.18^a^	2.31 ± 0.26^b^	1.58 ± 0.20^a^

Values are expressed as the means ± SEs (*n* = 3); different letters represent statistically significant differences (*P* < 0.05). ^1^SFA: sum of saturated fatty acids. ^2^MUFA: sum of monounsaturated fatty acids. ^3^*n*−6 PUFA: sum of *n*−6 polyunsaturated fatty acids. ^4^*n*−3 PUFA: sum of *n*−3 polyunsaturated fatty acids.

**Table 9:** Effects of different dietary selenium sources on muscle textural characteristics of yellow catfish.

	Control	Na_2_SeO_3_	Se yeast	Se–SP
Skin strength (g)	813.32 ± 26.77	784.21 ± 23.07	804.09 ± 22.13	806.68 ± 17.46
Adhesiveness (g)	−400.5 ± 28.57	−331.86 ± 29.58	−372.08 ± 32.45	−373.00 ± 36.15
Firmness (g)	268.5 ± 10.81^a^	306.96 ± 9.70^b^	300.30 ± 6.71^b^	293.04 ± 5.86^b^
Springiness (%)	50.30 ± 1.41^a^	54.39 ± 1.00^b^	50.41 ± 0.95^a^	52.62 ± 1.13^ab^
Flexibility (mm)	3.42 ± 0.12^a^	4.06 ± 0.17^b^	3.48 ± 0.09^a^	3.59 ± 0.14^a^
Fracturablity (g/s)	30.06 ± 1.27^a^	25.39 ± 0.84^b^	29.13 ± 0.72^b^	28.56 ± 1.11^b^
Stringiness (mm)	2.64 ± 0.10^a^	3.26 ± 0.18^b^	2.68 ± 0.12^a^	3.00 ± 0.10^ab^
Stickiness (g)	3.62 ± 0.13^a^	4.37 ± 0.21^b^	4.13 ± 0.21^ab^	3.81 ± 0.23^ab^

Values are expressed as the means ± SEs (*n* = 6); different letters represent statistically significant differences (*P* < 0.05).

**Table 10:** Effects of different dietary selenium sources on hepatic and plasma antioxidant enzymes of yellow catfish.

	Control	Na_2_SeO_3_	Se yeast	Se–SP
Liver				
GPX	69.39 ± 12.91^a^	85.15 ± 7.53^ab^	115.02 ± 12.71^b^	104.92 ± 10.20^b^
GR	5.16 ± 0.89	6.80 ± 1.21	6.95 ± 1.19	6.69 ± 1.22
SOD	239.59 ± 11.71	223.58 ± 12.79	236.54 ± 13.43	262.60 ± 13.09
CAT	449.75 ± 25.81^a^	558.49 ± 33.3^ab^	606.57 ± 90.28^ab^	751.87 ± 91.14^b^
MDA	1.58 ± 0.12^b^	0.99 ± 0.33^a^	0.80 ± 0.05^a^	1.04 ± 0.11^a^
Plasma				
GPX	195.43 ± 16.10^a^	187.26 ± 10.72^a^	251.53 ± 11.21^b^	183.89 ± 13.82^a^
SOD	166.75 ± 9.42	173.84 ± 11.05	182.10 ± 8.92	185.09 ± 8.50
MDA	10.71 ± 0.69	9.96 ± 0.82	10.76 ± 0.72	11.85 ± 0.91

GPX, glutathione peroxidase, U/mg protein in liver and U/mL in plasma; GR, glutathione reductase, U/g protein; SOD, superoxide dismutase, U/mg protein in liver and U/mL in plasma; CAT, catalase, U/mg protein in liver and U/mL in plasma; MDA, malondialdehyde, nmol/mg protein in liver and nmol/mL in plasma. Values are expressed as the means ± SEs (*n* = 6); different letters represent statistically significant differences (*P* < 0.05).

## Data Availability

The data that support the findings of this study are available from the corresponding author upon reasonable request.
